# Nutrition Intervention Using Behavioral Change Communication without Additional Material Inputs Increased Expenditures on Key Food Groups in Bangladesh

**DOI:** 10.1093/jn/nxz339

**Published:** 2020-01-14

**Authors:** Andrea M Warren, Edward A Frongillo, Phuong H Nguyen, Purnima Menon

**Affiliations:** 1 University of South Carolina, Columbia, SC, USA; 2 Poverty, Health, and Nutrition Division, International Food Policy Research Institute, Washington, DC, USA

**Keywords:** behavioral change communication, nutrition, household expenditures, food expenditures, dietary diversity, maternal and child nutrition, Bangladesh

## Abstract

**Background:**

Behavioral change communication (BCC) promotes skills and knowledge to improve infant and young child feeding, but without additional material inputs, recipients must develop strategies to translate knowledge into action. Using data from the Alive & Thrive initiative in Bangladesh (2010–2014), we aimed to test whether households receiving the intensive intervention (opposed to the nonintensive intervention) increased expenditures on key foods for mothers and children (e.g., foods that were promoted by the intervention and also changed in maternal and child diets).

**Methods:**

The intensive intervention provided interpersonal counseling, community mobilization, and mass media campaigns to promote breastfeeding and complementary feeding. A cluster-randomized design compared 20 subdistricts randomly assigned to the intensive (4281 households) or nonintensive (4284 households) intervention. Measures included food and nonfood expenditures, dietary diversity, and women's economic resources. Linear and logistic regression tested difference-in-differences (DD) in expenditures and dietary diversity, accounting for subdistricts as clusters, and the association between maternal and child consumption of specific food groups and corresponding food expenditures.

**Results:**

Expenditures on eggs and flesh foods increased more in intensive areas than in nonintensive areas by 53 (*P* < 0.01) and 471 (*P* < 0.01) taka/mo, respectively. Household food expenditures increased more in intensive areas by 832 taka (*P* = 0.02), whereas changes in nonfood expenditures did not differ. Women's employment and control of income increased more in intensive areas by 12 (*P* = 0.03) and 13 (*P* < 0.01) percentage points, respectively, while jewelry ownership decreased more by 23 percentage points (*P* < 0.01). Higher expenditures on food groups were reflected in higher consumption by women and children.

**Conclusions:**

Recipients in the intensive intervention mobilized additional resources to improve diets, reflected in increased expenditures and consumption of promoted foods. BCC interventions should document how recipients produce desired results without additional material inputs, particularly for behaviors that likely require additional resources. This trial was registered at clinicaltrials.gov as NCT01678716.

## Introduction

Behavioral change communication (BCC) refers to the use of communication strategies to promote the sustained adoption of a desired health behavior or behaviors that may lead to positive health outcomes. Common means of BCC include interpersonal counseling, print and virtual educational materials, and mass media campaigns (1). Nutrition-specific and -sensitive interventions in low- and middle-income countries that facilitate access to services or material inputs (e.g., medical care, cash transfers, livelihood programs) often implement complementary BCC programming. In this context, BCC has been shown to enhance program uptake and increase positive nutrition outcomes (2–4).

A review of 18 BCC-only interventions to improve nutrition in low- and middle-income countries found that impact was commonly assessed in terms of changes in knowledge, attitudes, and practices, but few BCC interventions posited explicit theories of change or program impact paths that would help to elucidate how or why impact was achieved (5). BCC promotes the skills and knowledge necessary for nutrition behavior change, but in the absence of complementary interventions that provide or facilitate material inputs to support new consumption and feeding behaviors, recipients must develop other strategies to translate knowledge into action. Achieving an adequate diet for both mothers and children, particularly in contexts of scarcity, is often a matter of both diversifying consumption and increasing consumption of key food groups, which typically requires additional material (e.g., money) and/or nonmaterial (e.g., time, knowledge) inputs. Theoretically, programs intended to improve the quality of diets of mothers and children through BCC must do so by shifting to mothers and children the intrahousehold distribution of food that is already there and/or increasing expenditures on food for mothers and children through reallocation or augmentation of funds. To our knowledge, only 1 study of a BCC-only intervention has assessed impact of the intervention on food expenditures to help understand how the BCC materially translates into dietary change. A cost-effectiveness study of the Expanded Food and Nutrition Education Program in the United States demonstrated that the program promoted greater monetary savings in food expenditures than it cost to implement (6). The program also promoted greater nutrient intake and reduced the incidence of running out of food on a monthly basis among recipients.

The Alive & Thrive initiative in Bangladesh consisted of a multipronged BCC strategy aimed at improving infant and young child feeding practices that was delivered through interpersonal counseling, mass media, and community mobilization. The intensive intervention was intended to promote the purchase and consumption of animal-source foods, vitamin A–rich fruits and vegetables, iron-rich vegetables, pulses, and added oils and fried foods for pregnant women and children aged >6 mo and to reduce the consumption of sweetened beverages for children >6 mo old (7). The intensive (compared with the nonintensive) intervention had a positive impact on infant and young child complementary feeding practices in Bangladesh, including improvements in dietary diversity, minimum acceptable diet, minimum meal frequency, and consumption of iron-rich foods (8). Building on those findings, we aimed to elucidate how the BCC messages were translated into action by testing whether households in the intensive intervention, compared with the nonintensive intervention, increased expenditures on foods for mothers and children that were being promoted and changed in maternal and child diets. To address this aim, we posed 4 research questions:

1. Did food expenditures change more in the intensive areas compared with the nonintensive areas, and which food expenditures changed?
2. Were changes in nonfood expenditures different between the intensive and nonintensive areas?
3. How did changes in food expenditures correspond with changes in maternal and child diets?
4. Did differences between intensive and nonintensive areas in changes in mother's employment and economic resources correspond with the difference in changes in food expenditures?

## Methods

### Intensive intervention

A detailed description of the intensive intervention package of interpersonal counseling and community mobilization has been provided elsewhere (8–10). The Alive & Thrive intervention implemented 3 simultaneous BCC interventions (i.e., interpersonal counseling, community mobilization, and a mass media campaign) to promote breastfeeding and complementary feeding for infants and young children.

In nonintensive intervention areas, standard infant and young child feeding services were delivered through home visits and interpersonal counseling by pre-existing BRAC (a large nongovernmental organization based in Bangladesh) frontline health workers (Shasthya Kormi) and volunteers (Shasthya Sebika). In intensive intervention areas, participants received the standard service along with additional interpersonal counseling that was delivered by a new cadre of frontline workers focused on infant and young child feeding (Pushti Kormi), along with the Shasthya Sebika volunteers. The intensive version of the counseling delivered by Pushti Kormi consisted of age-targeted visits to pregnant women and mothers of children <2 y of age, along with coaching and engagement of other household members. Furthermore, in the intensive areas, the Shasthya Sebika volunteers received incentives for each mother who demonstrated that she had adopted the appropriate behavior. Frontline workers received extensive training and were closely supervised.

Interpersonal counseling for the intensive areas began in 22 subdistricts in August 2010 and in another 28 subdistricts in August 2011. The mass media campaign, consisting of 7 nationally televised spots, began in December 2010 and was intensified to reach national coverage by February 2011. For intensive areas in which access to electricity and/or television was low, video materials on infant and young child feeding were screened locally. Community mobilization began in August 2011. All intervention components were implemented through the end of 2014 in the intensive areas (8, 10).

### Study design and participants

**FIGURE 1:**
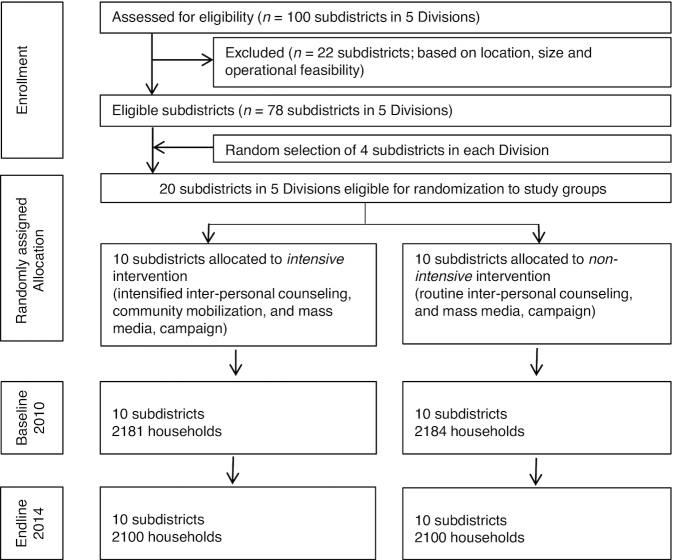
CONSORT diagram.

To compare intensive and nonintensive interventions, a cluster-randomized, nonblinded impact evaluation was used (9). Twenty subdistricts were randomly assigned to either the intensive or nonintensive intervention package (**Figure 1**). Cross-sectional household surveys were conducted at baseline (2010) and at endline (2014) in the same communities and in the same season. For this study, a total of 4281 households receiving the intensive intervention and 4284 households receiving the nonintensive intervention were surveyed at baseline or endline. Within each subdistrict, 5 unions and 2 villages within each union were randomly selected to yield a total of 200 villages, each with an average of 250 households. A household census was conducted at baseline and endline in each village to document mothers, infants, and infant date of birth. A list of all households with infants stratified by age group (<6, 6 to <24, and 24 to <48 mo) was created. Households were selected for surveys by using systematic sampling beginning with a random seed start point to yield the desired sample size per cluster. Mothers with an obvious mental disability that would prevent them from understanding and answering questions were excluded.

### Measurements

Food and nonfood expenditures were assessed at the household level using standard methodology and instrumentation (11). For food expenditures, the primary respondent was typically the female who was responsible for food management. If she did not know the price of an item, then the male who shopped for the item responded. For nonfood expenditures, both the female and male responded depending on their knowledge of the item. The expenditure module took 30–50 min to complete.

Food expenditures were assessed for 294 individual food items that were grouped into the following 15 categories for analysis: grains, legumes, dairy, flesh foods (total expenditures reported as well as disaggregated categories of meat, small fish, and big fish), eggs, vegetables, leafy vegetables, fruits, oils, spices, other foods, beverages, and foods prepared outside of the home. The recall period was 7 d, and data were collected on foods that were purchased, grown, or obtained from other sources, along with quantities consumed. Expenditure variables were constructed to reflect monthly equivalent expenditures assuming usual expenditure patterns by multiplying by 4.3.

Nonfood expenditures were assessed for 75 individual items across 19 categories. Recall periods were 1 wk for fuel, local transport, and a few miscellaneous items; 1 mo for utilities, house rent, health expenses, communication, personal items, entertainment, nonlocal travel, and a few miscellaneous items; and 12 mo for clothing, footwear, furniture, appliances, other household goods, taxes and fees, family events, education, vehicles, and a few miscellaneous items. All expenditures were constructed into monthly equivalents as a common unit. Monthly nonfood expenditures were categorized into nonlumpy (e.g., regularly occurring expenses such as fuel, utilities, education, and entertainment) and lumpy expenditures (e.g., relatively infrequent expenses or those not assumed to be regularly occurring, such as medical bills, weddings, funerals, and court expenses).

Maternal dietary diversity was assessed by using a list to ask about the types of foods eaten by the mother during the previous day and at night. The response options were “consumed” (scored 1) and “not consumed” (scored 0). Foods were organized into 9 groups: *1*) grains, roots, and tubers; *2*) legumes and nuts; *3*) dairy products (milk, yogurt, and cheese); *4*) flesh foods (meat, fish, poultry, and liver/organ meats); *5*) eggs; *6*) vitamin A–rich fruits and vegetables; *7*) leafy vegetables; *8*) other fruits; and *9*) other vegetables (12). The score for women is the sum of the responses to the 9 groups and ranged from 0 to 9. Child dietary diversity was assessed similarly as the sum of 7 food groups (8).

To assess women's employment, we used women's self-reported employment status (yes or no) and type of occupation. To assess economic resources, we used whether women had their own money to use (yes or no) and gold jewelry ownership (yes or no denoting sole or joint ownership of gold jewelry compared with no ownership) as a form of savings.

### Ethical approval

The institutional review boards at the International Food Policy Research Institute and the Bangladesh Medical Research Council both approved this study. All women were provided with detailed information about the study in writing and verbally at recruitment, and written informed consent was obtained. This trial was registered at clinicaltrials.gov as NCT01678716.

### Data analysis

The intensive intervention was intended to differentially increase expenditures on legumes, eggs, flesh foods, dairy, and fruits and vegetables (8) (i.e., increase these expenditures in the intensive areas compared with the nonintensive areas). Expenditures in 2010 were adjusted to 2014 taka using an inflation rate of 37.6% (13). Both aggregated and disaggregated food and nonfood expenditures were examined to determine which components differentially increased or decreased. Accounting for subdistricts as clusters, regression models were used to test the difference-in-differences (DD) (i.e., the differences between intervention areas in the changes from baseline and endline). The models specified terms for intervention area, year, and their interaction, with the latter estimating the DD (14). Differences in changes over time between intensive and nonintensive areas were also expressed as a percentage of the average starting values, calculated as the DD divided by the mean of the baseline value between areas multiplied by 100. Linear regression was used for expenditures, with the exception that some analyses were done with Tobit models to account for zero expenditures (15). Box-Cox transformations were used for distributions that were highly skew. Linear regression was used to test the DD in overall maternal dietary diversity and logistic regression for food groups. Logistic regression was used to test the association between maternal and child consumption of specific food groups and corresponding food expenditures. *P* values <0.05 were considered significant. Statistical analysis was conducted with Stata 15 (StataCorp).

For both expenditures and maternal and child dietary diversity, we analyzed all households together. To check whether results differed by child age, given that children aged ≤48 mo were sampled, we stratified expenditures and maternal dietary diversity by child age group (0–6, 6–24, and >24 mo) and found essentially no differences across strata (results not presented).

The analyses just described allowed assessment of the correspondence of expenditures for households and consumption of mothers at the group level. To further assess the extent of correspondence of expenditures for households and consumption of mothers and children at the household level (i.e., to assess whether households with greater expenditures of a food group had mothers and children who consumed more of that food group), we regressed consumption for 2 food groups (eggs and flesh foods) on expenditures using logistic regression and quantified the magnitude of association using ORs and the prediction accuracy using the area under the receiver operating characteristic (ROC) curve (16). We selected which food groups to test based on whether they had been promoted by the intervention and significantly changed in both expenditures and diets. For comparison, similar analyses were done to assess the extent of correspondence of maternal and child consumption for the food groups for which expenditures increased; previous analyses on maternal and child dietary diversity showed that the number of food groups mothers consumed was strongly associated with the number of food groups that children consumed in this sample but did not test the association by food group (17). These analyses used pooled data from baseline and endline, intensive and nonintensive areas, and child age after first confirming that the associations were similar across these strata.

## Results

### Changes in food and nonfood expenditures

Monthly expenditures for eggs, flesh foods, and beverages increased significantly more in the intensive intervention areas than in the nonintensive areas (**Table 1**). The differences expressed as percentage increases were 28% for eggs, 27% for total flesh foods, and 54% for beverages. Monthly expenditures on other foods increased significantly less in the intensive intervention areas than in the nonintensive areas, with the difference as a percentage being 25%. Monthly total household food expenditures and monthly household expenditures including food and nonlumpy expenditures increased significantly more in the intensive areas than in the nonintensive areas (**Table 2**). The differences as percentage increases were 10% for both monthly food expenditures and food expenditures combined with nonlumpy expenditures. No differential changes were observed in lumpy and nonlumpy expenditures, alone or in combination.

**TABLE 1:** DD from baseline to endline in household food expenditures (2014 taka) by food type^1^

	Baseline		Endline			
	Intensive	Nonintensive	Intensive	Nonintensive	DD	*P*
Legumes	181	193	250	248	15	0.09
Eggs	138	154	238	201	53	<0.01
Flesh foods^2^	1686	1849	2811	2503	471	<0.01
Meat and poultry	623	686	1124	1086	102	0.07
Small fish	900	829	1442	1106	264	0.13
Big fish	163	335	245	311	106	0.31
Vegetables	860	905	1070	1047	68	0.23
Leafy vegetables	91	81	123	130	−17	0.48
Fruits	796	801	1053	907	152	0.18
Dairy	248	265	284	276	25	0.82
Oil	295	327	413	428	16	0.42
Grains	2722	2793	2234	2261	43	0.66
Sugar	123	107	107	114	−23	0.15
Salt	33	40	44	49	2	0.26
Spices	278	238	351	322	−12	0.75
Beverages^3^	83	138	132	67	120	<0.01
Prepared foods^4^	271	314	265	342	−34	0.17
Other foods^5^	175	199	191	264	−47	0.02

1 All expenditure distributions were highly skew; Box-Cox transformations were applied. Tobit models were used to account for zero expenditures for all categories except for grains, vegetables, oil, and spices. *P* values were obtained from Tobit models, with the exception of grains, vegetables, oil, and spices, for which *P* values were obtained from linear regression. DD, difference-in-differences.

2 Flesh foods include meats, poultry, and fish.

3 Beverages include prepared tea, soda, and packaged juice.

4 Prepared foods consist of 35 diverse items including rice, curry, dal, salad, sweets, and any fried foods.

5 Other foods include sugar, biscuits, and tea leaves.

**TABLE 2:** DD from baseline to endline in monthly HH food and nonfood expenditures (2014 taka) as well as mothers’ employment and economic resources^1^

	Baseline		Endline			
	Intensive	Nonintensive	Intensive	Nonintensive	DD	*P*
Monthly HH food expenditure	7978	8404	9565	9159	832	0.02
Monthly nonfood expenditures (lumpy plus nonlumpy)	6355	7141	6850	7429	207	0.63
Monthly HH expenditures^2^	11,219	12,059	13,535	13,250	1125	0.02
Monthly total HH expenditures^3^	14,296	15,501	16,398	16,568	1035	0.09
Mother is employed, %	5.05	7.36	27.24	17.48	12.07	0.03
Mother has own money to use, %	44.73	47.75	55.41	45.10	13.30	<0.01
Gold jewelry ownership by mother, %	76.23	61.07	73.27	81.00	−22.89	<0.01

1 DD, difference-in-differences; HH, household.

2 Combined food and nonfood expenditures (without lumpy expenditures).

3 Combined food, nonfood, and lumpy expenditures.

### Changes in mothers’ employment and economic resources

Mothers’ employment and mothers having their own money to spend increased significantly more in intensive areas than in nonintensive areas; gold jewelry ownership decreased significantly in intensive areas, whereas it increased in nonintensive areas (Table 2). At endline, the mother being employed significantly predicted the mother having control of her own money (OR: 2.6; *P* = 0.02). Nearly all of the difference in employment at endline in the intensive arm was accounted by the mothers being self-employed. Mothers of older children were slightly more likely to be employed than mothers of younger children, but the difference between intensive and nonintensive arms at endline was about the same for younger and older children (results not presented).

### Changes in maternal and child diets and correspondence with food expenditures

Maternal dietary diversity score in the nonintensive areas increased significantly from 4.66 to 4.92 and in the intensive areas from 4.55 to 5.25, for a DD of 0.44 food groups in favor of the intensive areas (*P* = 0.05). For specific food groups, the intake of eggs and flesh foods increased significantly more in the intensive areas than in the nonintensive areas (**Table 3**).

**TABLE 3:** DD from baseline to endline in mothers’ dietary consumption by food group^1^

	Baseline, %		Endline, %			
	Intensive	Nonintensive	Intensive	Nonintensive	DD, percentage points	*P*
Legumes and nuts	26.13	39.65	43.24	45.62	11.13	0.06
Eggs	22.28	23.58	38.76	32.95	7.12	0.05
Flesh foods	70.88	63.69	80.81	66.86	6.76	0.02
Other vegetables	88.67	94.09	93.57	92.43	6.56	0.07
Dark-green leafy vegetables	41.59	45.42	49.76	49.71	3.88	0.51
Vitamin A–rich fruits and vegetables	47.23	45.47	48.0	44.33	1.91	0.77
Other fruits	27.65	26.47	34.86	25.95	7.72	0.18
Dairy	30.12	27.34	36.14	34.67	−1.31	0.64

1 The group of cereals, roots, and tubers was not analyzed because only 5 mothers in the sample (1 at baseline and 4 at endline) did not consume foods in this group. *P* values were obtained from logistic regression models. DD, difference-in-differences.

Mothers’ and children's consumption of eggs and flesh foods were both significantly predicted by expenditures (**Table 4**). Mothers’ consumption of a food group was a stronger predictor of child consumption of that food group than were food expenditures. Eggs had a larger OR and prediction accuracy than flesh foods.

**TABLE 4:** Associations of 3 food groups between child food consumption, maternal food consumption, and food expenditures, with prediction accuracy quantified by the area under the ROC curve^1^

	Food expenditures (50 taka)^2^				Maternal consumption	
	Maternal consumption		Child consumption		Child consumption	
Predictor Outcome	OR	ROC area	OR	ROC area	OR	ROC area
Eggs	1.190	0.721	1.142	0.706	12.274	0.771
Flesh foods	1.025	0.714	1.006	0.602	9.264	0.692

1 Pooled baseline and endline and intensive and nonintensive for all values. Baseline expenditures were adjusted to 2014 taka. All ORs, *P* < 0.01. ROC, receiver operating characteristic.

2 ORs were estimated for 50-taka differences.

## Discussion

Expenditures on key food groups promoted by the intensive intervention increased more in that arm than in the nonintensive arm. Overall monthly food expenditures differentially increased by 832 taka/mo (*P* = 0.02). No differential changes occurred in lumpy and nonlumpy nonfood expenditures, suggesting that households were not reallocating substantial amounts of money from nonfood expenditures to food expenditures. The observed increases in expenditures were reflected in maternal and child dietary intake of these key food groups.

With no evidence of resource reallocation from nonfood to food expenditures within households (i.e., decreased regular and occasional nonfood expenditures), households may have needed to develop different strategies to bring in additional income to purchase key foods. Just 5% of women in the intensive intervention areas were employed at baseline as compared with 7% of women in nonintensive areas; at endline, 27% of women in the intensive areas were employed as compared with 17% in nonintensive areas (*P* = 0.027). The observed increase in maternal employment in the intensive intervention areas, in combination with the increased likelihood of a women being employed outside of the home to have control over the use of their income, suggest 2 key inferences about the functioning of the BCC intervention. First, women and families were motivated by the intensive intervention to prioritize the diets of women and children. Second, women took extra intervention steps to muster the additional resources required to achieve an adequate diet for themselves and their children. Taken together, these inferences provide a plausible explanation of how the intensive intervention worked to improve women's and children's diets. That is, the additional funds ostensibly generated through women's increased employment may have been put toward household food expenditures. This explanation is supported by findings regarding differences in jewelry ownership between the intensive and nonintensive intervention arms. Gold jewelry constitutes a form of tangible savings within a household, is typically considered to be a woman's asset in Bangladesh, and women commonly choose to invest their earned income in gold jewelry (18, 19). Women in the intensive areas reported increased prevalence of employment from baseline to endline (5–27%) and a slightly decreased prevalence of gold jewelry ownership (76–73%). In contrast, employment for women in the nonintensive areas increased less (7–17%) and jewelry ownership increased substantially (61–81%). We thus posit that the progressive acquisition of gold jewelry is desirable, especially when circumstances permit women to earn and control income, yet women in the intensive arm were motivated to spend their income on food rather than invest in this traditional form of savings.

The increase in maternal employment suggests that mothers’ time was reallocated from something else toward earning income, largely through self-employment. Although unintended negative consequences theoretically could have resulted from the reallocation of time, the intensive intervention (compared with the nonintensive intervention) improved mothers’ and children's diets (8) as well as children's language and motor development (20), suggesting that, for children, the effects of the intervention were positive even if mothers were more likely to be employed.

These findings are in line with compelling evidence from the Alive & Thrive nutrition-focused maternal BCC intervention delivered along with a standard antenatal care package that improved food security among pregnant and lactating women in Bangladesh without additional material inputs (21). Frongillo et al. (21) suggested that the intervention achieved improvements in food security via motivating the use of savings and the reallocation of existing resources to pay for improved diets and promoting knowledge about food security and nutrition among both mothers and fathers. Our study provides evidence of some of the other concrete actions initiated by intervention recipients to translate BCC learnings into dietary outcomes at the household level.

Food expenditures predicted maternal and child consumption of eggs and flesh foods, but maternal consumption was a stronger predictor of child consumption than were food expenditures. The measures of prediction accuracy were 0.602–0.771, which represent modest prediction accuracy given that the theoretical range for prediction accuracy as measured by the area under the ROC curve is 0.5–1.0 (16). These estimates of prediction accuracy likely understate the correspondence for usual consumption because maternal and child consumption of food groups was measured by 24-h recall and concordance was counted only when both consumed a food group on the same day. On the other hand, both maternal consumption and child consumption of food groups were measured by similar methods, which differed from that used to measure food expenditures. This methodological difference may have affected the prediction accuracy of maternal consumption relative to that of food expenditures.

Expenditures were measured comprehensively using standard methods with the recall period adjusted to be appropriate to the types of goods that were being purchased. For diet, the list-based recall period was 7 d to reduce reporting error, with extrapolation to 1 mo. Extrapolation makes the assumption that consumption in 7 d is consistent for the entire month.

This study provides evidence of how well-designed nutrition BCC interventions delivered through strong health- and community-based systems bring about behavior change and contributes to our understanding of the ways in which program recipients translate information into action. BCC interventions frequently have monetary or other resource implications for recipients to be able to translate knowledge and skills into action. BCC interventions are typically targeted to people who are resource constrained, but process and impact studies infrequently document shifts in resources, including time use (5). This study draws attention to the need for BCC interventions to document what is required on the part of program recipients to produce the desired benefits, particularly when the BCC intervention concerns behaviors for which additional resources are likely required, such as in the management of inadequate diets, chronic diseases, and obesity. Furthermore, research is needed on the conditions under which nutrition BCC-only interventions can succeed without additional material inputs, including dose, topic, BCC format, intervention context, strength of service delivery systems, recipient characteristics, and length of exposure.

## Notes

This study was funded by the Bill & Melinda Gates Foundation, through Alive & Thrive, managed by FHI 360. Additional financial support to the evaluation study was provided by the CGIAR Research Program on Agriculture for Nutrition and Health (A4NH), led by the International Food Policy Research Institute.

Author disclosures: The authors report no conflicts of interest.

This study was a secondary analysis of data collected from the first phase of Alive & Thrive, which ran from 2009 to 2015.

Abbreviations used: BCC, behavioral change communication; DD, difference-in-differences; ROC, receiver operating characteristic.
